# Pediatric Laryngopharyngeal Reflux in the Last Decade: What Is New and Where to Next?

**DOI:** 10.3390/jcm12041436

**Published:** 2023-02-10

**Authors:** Valentinos Sofokleous, Anna-Maria Papadopoulou, Evangelos Giotakis, Alexander Delides, Efthymios Kyrodimos, Pavlos Maragoudakis, Ioannis Psarommatis

**Affiliations:** 1 Department of Pediatric Otorhinolaryngology, Athens Children’s Hospital “P. & A. Kyriakou”, 115 27 Athens, Greece; 2 1st Department of Otorhinolaryngology—Head & Neck Surgery, University of Athens, ‘Hippocration’ General Hospital, 115 27 Athens, Greece; 3 2nd Department of Otorhinolaryngology—Head & Neck Surgery, University of Athens, ‘Attikon’ University Hospital, 124 62 Chaidari, Greece

**Keywords:** pediatric, laryngopharyngeal reflux, extra-esophageal reflux, gastroesophageal reflux, infants, children

## Abstract

Background: Laryngopharyngeal reflux may affect people of any age; still, most of the accumulated knowledge concerns adults, and evidence regarding pediatric populations remains relatively restricted. This study aims to review the most recent and emerging aspects of pediatric laryngopharyngeal reflux from the last ten years. It also attempts to identify gaps in knowledge and highlight discrepancies that future research should urgently address. Methods: An electronic search of the MEDLINE database was conducted, limited to January 2012 through December 2021. Non-English language articles, case reports, and studies that concerned a purely or predominantly adult population were excluded. The information from the articles with the most relevant contribution was initially categorized by theme and subsequently synthesized into a narrative form. Results: 86 articles were included, of which 27 were review articles, eight were surveys, and 51 were original articles. Our review systematically maps the research done in the last decade and provides an updated overview and the current state-of-the-art in this subject. Conclusions: Despite discrepancies and heterogeneity in accumulating research, evidence gathered so far endorses a need for refining an escalating multiparameter diagnostic approach. A step-wise therapeutic plan appears to be the most reasonable management approach, starting with behavioral changes for mild to moderate, uncomplicated cases and escalating to personalized pharmacotherapy options for severe or nonresponsive cases. Surgical options could be considered in the most severe cases when potentially life-threatening symptoms persist despite maximal medical therapy. Over the past decade, the amount of available evidence has been gradually increasing; however, its strength remains low. Several aspects remain markedly under-addressed, and further adequately powered, multicenter, controlled studies with uniformity in diagnostic procedures and criteria are urgently needed.

## 1. Introduction

Laryngopharyngeal reflux (LPR) is defined as a retrograde migration of gastroduodenal contents that move beyond the upper esophageal sphincter to reach the pharynx and larynx [1,2]. Other terms used for the same condition are “extra-esophageal reflux”, “silent reflux”, and “supraesophageal reflux”. Although regarded in the past as a particular subtype of gastroesophageal reflux (GER), pathophysiologically, it is nowadays considered distinct from GER, which is defined as “the passage of gastric contents into the esophagus, with or without regurgitation or vomiting” [3].

LPR and GER are physiologic phenomena occurring transiently in healthy subjects. However, GER is considered non-physiologic and referred to as gastroesophageal reflux disease (GERD) when reflux leads to symptoms, such as heartburn and regurgitation, or complications, such as esophagitis and esophageal strictures, that impact a patient’s daily life. Likewise, LPR may be considered pathologic and referred to as laryngopharyngeal reflux disease (LPRD) when leading to troublesome laryngopharyngeal symptoms or causing or exacerbating related conditions. LPRD may affect any age; still, most of the accumulated knowledge concerns adults, and evidence regarding pediatric populations remains relatively restricted. Similar to adults, clinical hallmarks of GERD, such as heartburn or esophagitis, are only occasionally present in children with LPRD, and this may be why the disease is not unusually overlooked by otolaryngologists and pediatricians.

In the pediatric setting, reflux events appear to be most frequent during infancy, primarily due to the developmental immaturity of both the upper and lower esophageal sphincters [4,5]. Fortunately, most infants do not develop any associated feeding or airway problems that require further assessment or intervention, and most grow out of this condition by the end of their first year. However, a minority will, at some stage, present with symptoms or complications that range from mild to severe or even life-threatening. Furthermore, various disorders affecting preeminently children, such as laryngomalacia, subglottic stenosis, vocal fold nodules, dental erosion, pediatric chronic sinusitis, paradoxical vocal fold movement disorder, eustachian tube dysfunction, recurrent or chronic otitis media, adenotonsillar hypertrophy, asthma, recurrent pneumonia, bronchiectasis, and recurrent respiratory papillomatosis may be related to LPR, but a definite causal association has not been established in pediatric populations yet [6,7,8,9,10,11,12,13,14,15,16,17,18].

Although LPR has been recognized as a probable cause or complicating factor for numerous upper aerodigestive complaints and disorders, diagnosing children is still challenging and surrounded by controversy and disagreement. Up until now, there is no ideal test that can be considered the unequivocal gold standard for pediatric LPR detection. Concerning its management, there is a lack of clear guidance regarding the appropriate agents, dosage, duration, and safety of long-term use in the various pediatric age groups [19].

This study aims to review the most recent and emerging aspects of pediatric LPR from the last ten years regarding the epidemiology, pathophysiology, diagnosis, clinical spectrum, and management of the disease from an otorhinolaryngological point of view. In addition to providing an updated overview of the subject by systematically mapping the most recent research done in this area, this review also attempts to identify existing gaps in knowledge and highlight discrepancies and disputed aspects that future research should pressingly address.

## 2. Materials and Methods

Using relevant terms and Boolean logic, we conducted an electronic search of the MEDLINE bibliographical database in July 2022, retrieving studies from a 10-year period from January 2012 to December 2021. The following search strategy in the PubMed Search Builder was used: “((laryngopharyngeal reflux) OR (extra-esophageal reflux) OR (extraesophageal reflux) OR (supraesophageal reflux) OR (LPR) OR (LPR diagnosis) OR (LPR treatment)) AND ((children) OR (infants) OR (pediatric))”. All co-authors agreed upon the study protocol.

Working independently, three investigators (V.S., A.-M.P, and E.G.) searched the literature for eligible studies. Only articles in English were considered eligible. All titles of interest were further reviewed by abstract, while articles without abstracts were excluded. Case reports, in vitro studies, and animal studies were also excluded. All prospective and retrospective studies, as well as surveys and literature reviews, were considered eligible for full-text reviewing, except if, from their title or abstract, it was evident that they concerned a purely adult population. Original articles that concerned a mixed population were qualified for full-text evaluation. However, after full-text evaluation, some of them were subsequently excluded if they proved to concern predominantly or exclusively an adult population or if they did not fit into the conceptual framework of the study. We resolved disagreements on study selection by consensus and discussion with the rest of the authors if needed. Data charting and critical analysis of the literature were then performed by grouping articles according to their main focus, i.e., whether they addressed epidemiological, pathophysiological, clinical presentation, diagnostic, or treatment inquiries concerning LPR in the pediatric population. The three authors (V.S., A.-M.P., and E.G.) independently charted the body of evidence and discussed the results with the rest of the authors. Ethics committee approval was not required for this review.

## 3. Results

**Figure 1:**
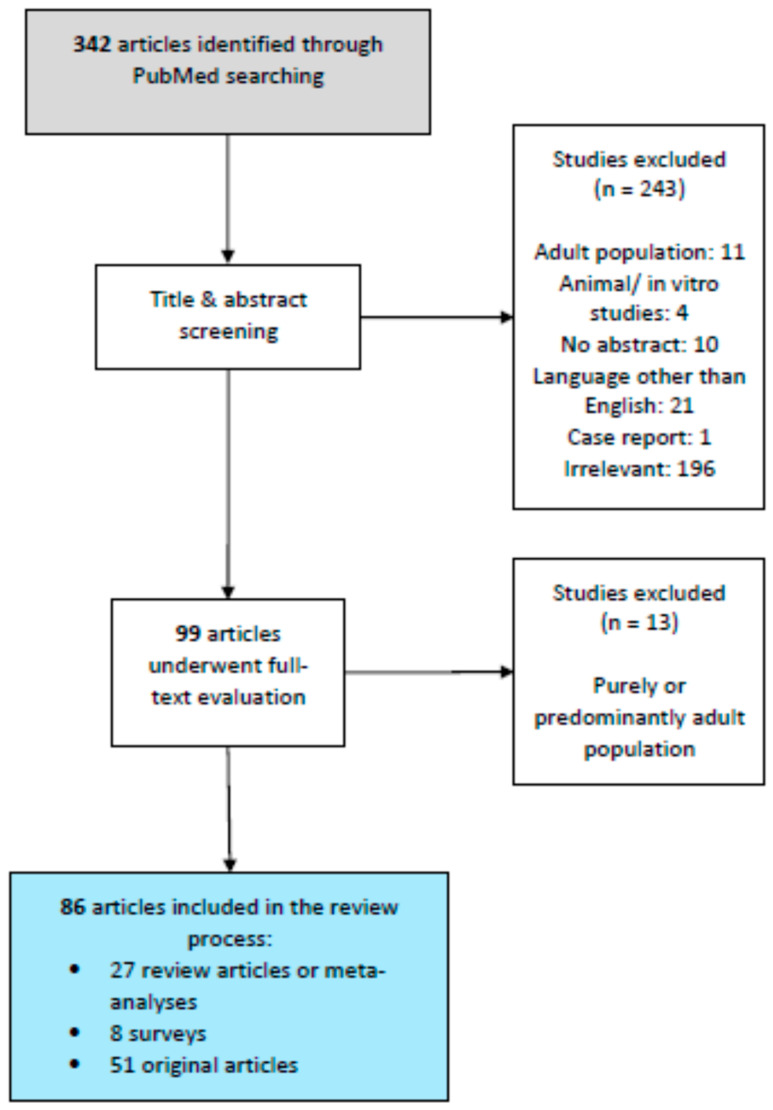
Flow diagram summarizing our search and selection strategy.

Our search strategy yielded 342 articles in total that were subsequently screened by title and abstract. Out of these articles, 21 were excluded as they were published in a language other than English, and ten were excluded because they included no abstract. A further 11 articles were excluded since—from their title or abstract, it was evident that they concerned a purely adult population. Another four studies were excluded as they constituted animal or in vitro studies, and one was further excluded as it concerned a report of two cases. Lastly, 196 articles were excluded as they were irrelevant to the scope of this article. In the end, 99 articles were selected for full-text review. Of these 99 articles, 13 were eventually excluded from further analysis after full-text evaluation as they concerned purely, or predominantly, an adult population, leaving 86 articles that were finally included as relevant to the scope of this review. Figure 1 summarizes our search and selection strategy.

Out of these 86 articles, 27 were relevant review articles, 8 were surveys, and the remaining 51 were original articles (Table 1); the latter studies were prioritized. Table 1 summarizes the most important features of the original articles concerning pediatric LPR that were published during the past decade. Additionally, important contemporary or earlier articles were manually selected from the reference lists of these 86 studies to better delineate some prior core knowledge and concepts and to allow a better appreciation of the advances in the field over time. The information from the articles with the most relevant contribution was initially categorized by theme and subsequently synthesized into a narrative form.

### 3.1. LPR Versus GER

LPR was first introduced as a clinical entity by Koufman et al. [60] more than three decades ago; however, the subject remains insufficiently illuminated in the medical literature. There is enough clinical evidence to suggest that LPRD is an entity quite separate from GERD; nevertheless, these two conditions frequently coexist. The causative defect in GER is believed to be lower esophageal sphincter dysfunction and dysmotility of the lower esophagus. By contrast, the primary defect in LPR is upper esophageal sphincter dysfunction [61]. These two entities display different reflux patterns, with GERD patients experiencing prolonged and frequent periods of reflux, while in LPRD, shorter or infrequent episodes may still harm the laryngopharyngeal mucosa, which is much more vulnerable to the detrimental effects of acid and enzymes. The intrinsic esophageal defense mechanisms against gastric acid are normally able to withstand dozens of distal reflux events and long periods of acid exposure. On the contrary, even one transient episode of proximal reflux into the laryngopharynx can probably induce laryngeal or pharyngeal symptoms [62].

Moreover, LPR patients experience episodes of reflux predominantly in the daytime and in the upright position as the upper esophageal sphincter is contracted when in the supine position and relaxes in the upright position [63,64]. In contrast, GER patients suffer predominantly supine/nocturnal episodes. Furthermore, GER and LPR display different symptomatology and pharmacological treatment responses [1,2,22,65]. These patterns are easily recognizable in adults and older children alike. In infants and neonates; however, it is often challenging to differentiate GERD from LPRD, as infants with GERD typically present with extra-esophageal symptoms. Furthermore, regurgitation, which is essentially LPR as stomach contents are refluxing all the way up to the oral cavity, is the most common symptom of GERD in this age group [66]. In infants and neonates, therefore, the terms are often used interchangeably.

### 3.2. Epidemiology

Although the prevalence of GER in the general population is reported to be 7.2%, no sufficient data exist on the prevalence of LPR, mainly due to the lack of a gold standard ensuring LPR diagnosis. However, it is estimated that up to 10% of adult patients presenting to ENT clinics may have complaints or conditions that can be attributed to reflux events affecting the laryngopharynx [60]. In the pediatric population, things are even more obscured, but the prevalence of pediatric reflux disease (LPRD/GERD) is believed to be even higher, affecting as much as 20% of children [5].

Infants are particularly prone to reflux disease, and episodes peak at 4–5 months of life [4]. It is believed that more than 50% of normal infants have regurgitation, which spontaneously resolves as they approach the age of one [67]. In most of these infants, LPR is considered a physiologic condition as they do not develop any associated feeding or airway problems that require further assessment or intervention, and most grow out of this condition by the end of their first year [31,68]. However, pediatric LPR may often go undiagnosed, with symptoms being attributed to other common pediatric conditions, such as upper respiratory infections or allergies. Currently, there is a pressing need for large-scale epidemiologic studies that take into account not only LPR signs and symptoms but also objective tests, such as combined multichannel intraluminal impedance-pH (MII-pH) monitoring or 24-h pharyngeal pH monitoring, to ascertain pediatric LPR prevalence.

### 3.3. Pathophysiology

The increased incidence of reflux events in neonates and young infants is believed to be associated with many factors, including the fact that they remain in a recumbent position for most of their day, their milk-based diet, and also both structural and functional differences, particularly the shorter length of the esophagus, the immaturity of both the upper and lower esophageal sphincters, as well as the lack of acute angle at the gastroesophageal junction [68,69]. Known risk factors for pediatric GER, including premature birth, neurologic conditions, repaired esophageal atresia, esophageal achalasia, hiatal hernia, obesity, and chronic respiratory disorders, may also act as risk factors for clinically overt pediatric LPR [5,29,70,71].

LPR can lead to upper respiratory tract irritation and inflammation via several mechanisms. The most important mechanism is a direct noxious effect of acidic gastric contents causing mucosal microtrauma and swelling, mucus hypersecretion, ciliary dyskinesia, and increased expression of inflammatory mediators. The mucosa of the laryngopharynx is much more vulnerable to acid exposure than the esophageal mucosa; pH-monitoring studies in adults suggest that up to 50 episodes of reflux into the esophagus in a day with a drop of pH below 4 are considered normal and are usually unable to cause microinjury and disease. However, even the shortest exposure of the laryngopharyngeal mucosa to gastric refluxate has been reported to have clinically relevant implications [1,2,60].

Recent research findings point toward the harmful effect of non-acidic reflux on the mucosa of the upper aerodigestive tract [72]. Findings show that in children, and especially in infants, many reflux episodes are non-acidic or weakly acidic [73,74,75] and that these events may also cause morphologic upper aerodigestive tract changes and laryngopharyngeal symptoms [36,39,75,76,77,78]. Pepsin, bile salts, and trypsin are thought to mediate these changes in non-acidic or weakly-acidic reflux [79,80]. Pepsin, in particular, is activated at a pH below 4.0 and remains active up to pH 6.5. In more alkaline environments, it is inactivated but is still stable and can be reactivated if the pH drops again, i.e., with the next acidic laryngopharyngeal reflux event. Recent studies show that pepsin can also be reactivated within the acidic intracellular environment after receptor-mediated uptake of pepsin by laryngeal epithelial cells, even if the pH in the throat is up to 7.4 [81]. Moreover, the laryngopharyngeal mucosa is still susceptible to pepsin even in a non-acidic environment because inactive pepsin can still stimulate the expression of many proinflammatory cytokines and receptors such as CXCL14, CCL20, IL1A, IL5, IL8, CCR6, CCL26, BCL6, and IL1F10 [82]. However, data relating symptoms and signs to non-acidic or weakly acidic reflux are rather heterogeneous and conflicting [25,56]. Undoubtedly, the importance of weakly acidic reflux in pediatric LPR needs further investigation, given its critical implications in the pharmacological management of pediatric LPR.

Another probable pathophysiologic mechanism that entails both pepsin and acidic reflux and also a compromised mucosal response to noxious stimuli concerns the reduced expression of carbonic anhydrase III (CA III) enzyme by the laryngeal mucosa. CA-III is habitually present in the normal laryngeal epithelium but is depleted following repeated exposure to pepsin, resulting in an inability of the laryngeal epithelium to produce sufficient bicarbonates to neutralize gastric acid upon contact with refluxate, thus predisposing the laryngeal mucosa to acidic reflux-related inflammatory changes [83,84].

A further recently elucidated mechanism consists of a compensatory vagus nerve neuroflexive response triggered by the decrease of pH in the distal esophagus, which may lead to bronchial or laryngeal spasm [2,85]. Interestingly, this mechanism, along with reflux-induced lung injury and hyper-responsiveness triggered by lower airway aspiration of refluxates, compose the most probable pathophysiologic mechanisms by which reflux disease can cause chronic or recurrent lower respiratory tract disease or aggravate asthma [20,57]. Another controversial theory postulates a supplemental contributing role for Helicobacter pylori [24,42,50,86]. Mucosa-associated lymphoid tissue in the Waldeyer ring is similar to lymphoid tissue of the stomach, which can be very favorable for the survival of H. pylori that further promotes inflammation, and also contributes to the reactive hyperplasia of the lymphatic tissue in the nasopharynx and tonsils [35,49].

### 3.4. Symptoms

The clinical spectrum of pediatric LPR varies considerably from mild complaints, such as a frequent need to clear the throat, to life-threatening apneic spells. Most patients with LPR do not show classic symptoms of GER, notably esophageal burning or heartburn. Nevertheless, they often present with a diversity of nonspecific symptoms from the pharynx and larynx due to the vulnerability of the upper-airway epithelium to the noxious gastroduodenal refluxate.

**Figure 2:**
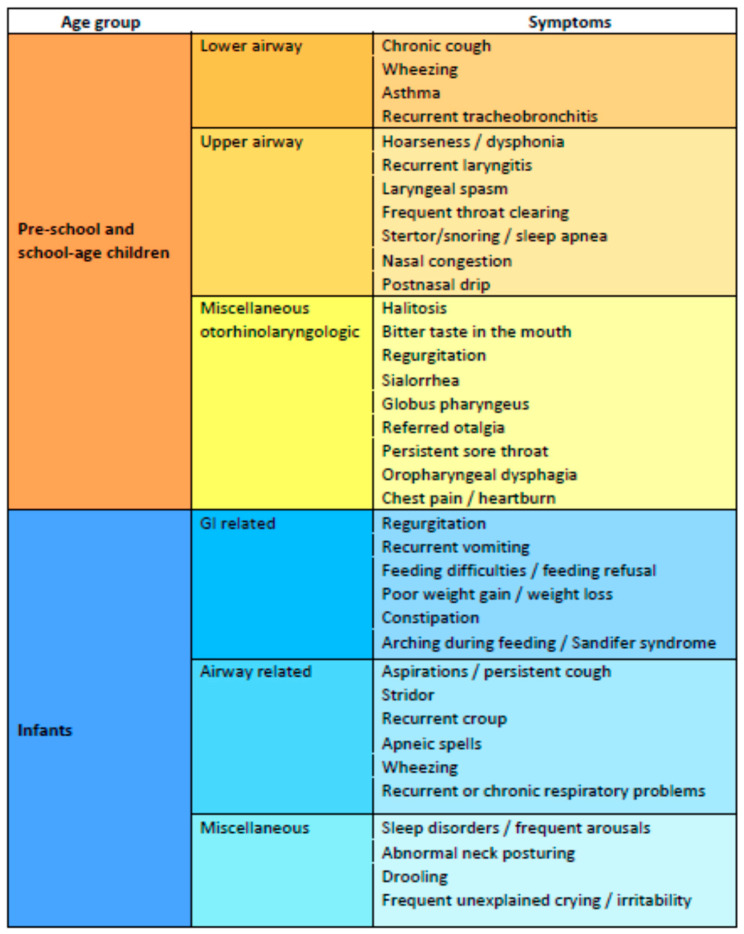
Non-exclusive list of symptoms of pediatric LPR.

Common symptoms of pediatric LPR in school-age children are similar to those in adults and are listed in Figure 2. Typical reflux symptoms can be assessed reliably in children older than eight years of age, as by that age, they can communicate regular symptoms more effectively [3,70]. Characteristic symptoms include a globus sensation, dysphonia, nasal congestion and postnasal drip, repetitive throat clearing, persistent sore throat, chronic cough, halitosis, sialorrhea, stertor or sleep apnea, recurrent laryngotracheitis, laryngeal spasm, paradoxical vocal fold movement, and dysphagia [2,47,68]. Importantly, infants and neonates may present with even vaguer signs and symptoms (Figure 2), including arching during feeding, irritability with frequent unexplained crying, feeding difficulties, poor weight gain, stridor, wheezing, apneic events, sleep disorders and frequent arousals, and recurrent respiratory problems [31,87,88]. The reported rate of clinical manifestations varies considerably among the reviewed studies, presumably because of the heterogeneity in the age of the study group, the method used to establish the diagnosis of LPR, and the existence of conditions causing symptoms similar to those of LPR.

Taking a thorough medical history, including the age of onset of symptoms, feeding habits, length of feedings, the volume of feeds, time interval between feedings, the pattern of regurgitation (e.g., nocturnal, postprandial, or long after meals), and performing a careful physical examination are all of paramount importance to distinguish LPR from LPRD, to identify possible complications of LPRD, and to exclude more worrisome disorders requiring further investigation and management. Nevertheless, the highly nonspecific nature of these symptoms across the whole pediatric age range also highlights the importance of thorough medical history and physical examination by all involved specialists (pediatricians, neonatologists, pediatric otolaryngologists, and pediatric gastroenterologists) prior to pursuing invasive diagnostic assessments.

### 3.5. Endoscopic Findings

**Figure 3:**
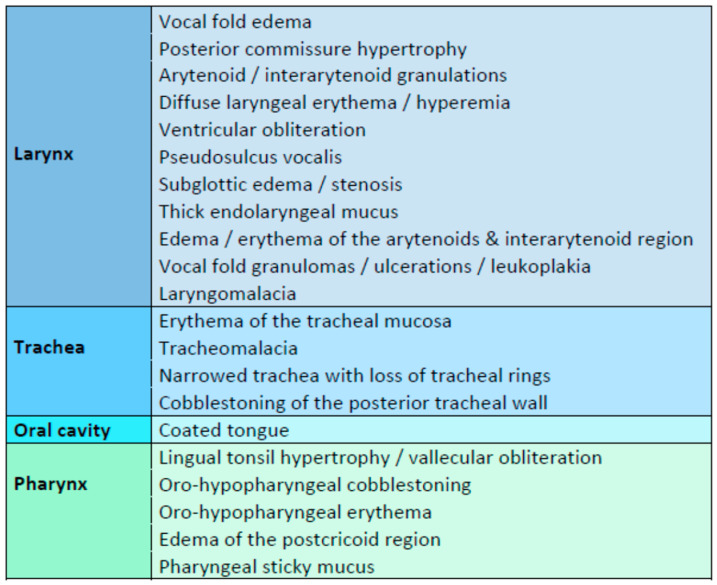
Non-exclusive list of endoscopic findings of pediatric LPR.

Flexible nasopharyngolaryngoscopy is crucial in assessing any pediatric patient with suspected LPR. It may identify various pharyngolaryngoscopic findings suggestive of LPR (Figure 3) or facilitate differential diagnosis by detecting alternative diagnoses or associated diseases, such as vocal folds nodules, recurrent juvenile papillomatosis, laryngeal granulomas or polyps, vocal fold palsy, subglottic stenosis, sulcus or pseudosulcus vocalis, laryngeal cleft, and laryngomalacia [27,89]. The most common pharyngolaryngoscopic findings of pediatric LPR, according to the reviewed studies, are presented in Figure 3. Among them, posterior commissure hypertrophy, arytenoid/interarytenoid erythema and edema, vocal fold edema, pharyngeal cobblestoning, and lingual tonsil hypertrophy were found to be important indicators of pediatric LPR [27,32,36,47,90,91].

In a recent, novel study, Galli et al. [31] explored the utility of Narrow Band Imaging (NBI) endoscopy in assessing pediatric LPRD. The NBI technology can detect neoangiogenesis and hypervascularization of the upper aerodigestive tract mucosa, which characterizes chronic inflammation. Their study claims that, compared to classic white light endoscopy, NBI ensures greater precision and efficacy in highlighting LPR-associated signs such as mucosal hyperemia, edema of the retrocricoid region, and cobblestone appearance of the hypopharynx. In the infant subgroup, it facilitated the detection of supposedly highly specific age-related signs, such as neovascularization and microerosions of posterior subglottic and tracheal mucosa and cobblestoning in the posterior wall of the trachea. However, conclusions are restricted by omitting a uniform use of an objective diagnostic tool, such as MII-pH, and the absence of healthy controls. Nevertheless, the results represent an interesting starting point for further studies and highlight the potential utility of NBI endoscopy in pediatric LPR evaluation.

### 3.6. Assessment—Diagnostic Tools

Diagnosing pediatric LPR is a big challenge, and an unequivocal diagnostic algorithm is currently unavailable. As has already been stressed, most symptoms and physical findings of pediatric LPR are vague and nonspecific and, on their own, may be insufficient for diagnosis because a similar clinical picture can derive from infections, allergies, postnasal drip, exposure to toxic inhalants, passive smoking, and chronic inflammation of other etiology. Clinicians need to combine reported symptoms and subjective findings with objective examinations, such as pharyngeal 24 h pH monitoring or MII-pH monitoring for accurate diagnosis [25]. On the other hand, (along with objective, instrumental tools) typical symptoms and signs and their burden on a child’s daily life should always be sought and evaluated since they are required to make a distinction between LPR, which may be a physiologic condition, and (non-physiologic) LPRD. However, objective testing is costly, invasive, and uncomfortable, and its employment in daily practice and outside the research setting is not always meaningful [40]. Especially in infants and younger children, the objective/instrumental investigation may be appropriate only in the presence of warning signs, if there is an impact of the symptoms on normal growth or acquisition of developmental milestones, or when the initiation of pharmacotherapy is to be considered, as mild and typical cases usually respond well to dietary and behavioral modifications alone [3]. The following instrumental and non-instrumental diagnostic tools are the ones most commonly used:

#### 3.6.1. Reflux Symptom Index (RSI), Reflux Finding Score (RFS), and Other Clinical Scoring Systems

The Reflux Symptom Index (RSI) is a patient-reported outcome measure designed and validated in the adult population by Belafsky et al. RSI scores greater than 13 are considered suggestive of LPR in the adult population [92]. Such symptoms can be assessed reliably in children 8–12 years old or older [3]; however, the questionnaire has not been validated for use in the pediatric age group. Furthermore, it does not consider many LPR symptoms that are presumably more frequent at younger ages, such as ear pressure, poor weight gain, arching during feeding, recurrent croup, and halitosis. It also assesses the severity of relevant symptoms but does not take into consideration the frequency or duration of these symptoms.

The Reflux Finding Score (RFS) is used to evaluate endoscopic findings of the larynx. This tool comprises eight parameters, each with a specific scoring method. It assesses the presence and severity of findings, such as subglottic edema, ventricular obliteration, erythema/hyperemia, vocal fold edema, diffuse laryngeal edema, posterior commissure hypertrophy, granuloma, and accumulation of thick endolaryngeal mucus [93]. RFS scores greater than 7 are accepted as suggestive of LPR. However, several studies showed that the RFS score is generally lower in pediatric patients compared to adults with LPR, and a reduced cut-off point was proposed for increased test specificity in the pediatric population [32,36].

Research findings repeatedly showed that neither RSI nor RFS is accurate enough in predicting LPR in infants and children [25,94]. In children, the evaluation of the larynx with flexible laryngoscopy might be hampered by the small-scaled anatomic landmarks and thinner endoscopes with lower image resolution. Secondly, awake and agitated young children and infants provide limited visibility of details in the examination videos [48]. In children, important extra-laryngeal endoscopic signs of reflux not included in the RFS are lingual tonsil swelling, anterior pharyngeal pillar inflammation, a coated tongue, oro- and hypo-pharyngeal cobblestoning, and tracheal inflammation, or narrowed trachea with loss of tracheal rings (Figure 3) [31].

Based on the RFS, Van der Pol et al., in 2014, designed the Reflux Finding Score for Infants (RFS-I), with scores ranging from 0 to 8. However, only moderate interobserver agreement was reached, with the highly variable intraobserver agreement [48]. Another recent study also supported the fact that the RFS-I is unsuitable for detecting signs of LPR in infants or guiding treatment, neither with flexible nor with rigid laryngoscopy [38]. Another attempt to create an assessment tool came from Baudoin et al. in 2014. They proposed a pediatric LRP diagnostic probability scoring tool that took into account relevant symptoms and local findings in oropharyngoscopy and fiberoptic endoscopy, as well as the presence of obesity or associated comorbidities (such as asthma or recurrent laryngitis) [47]. In 2020, Košec et al. created a new, elaborated form of this tool [28]. Both systems stratified patients into three grades, each with a different risk of LRP (low, moderate, or high risk for LPR). This could guide clinicians in defining groups that request further, more invasive evaluation, but their validity, usefulness, and cost-effectiveness remain to be proven.

#### 3.6.2. Multichannel Intraluminal Impedance-pH Monitoring (MII-pH)

Currently, MII-pH monitoring is considered by many investigators the diagnostic gold standard of LPR [2,89], as it detects both acidic refluxes, weakly acid reflux, and non-acidic reflux. It may also determine the refluxate’s composition (liquid, gas, or mixed) and the height reached by the refluxate [36]. MII can detect esophageal content movement by measuring changes in electrical resistance. A single-use, 2 mm thin catheter with ring electrodes positioned at various intervals along its length is placed inside the lumen of the esophagus and pharynx. Intraluminal content movement (liquid and gas) is detected as sequential changes in impedance along the catheter. Reflux episodes are manifested as a decrease in impedance of at least 50% of the initial value in the distal channels that proceed to the more proximal. Each reflux episode is then correlated to the pH reading at that time and is considered acidic if pH < 4, weakly acidic when the pH value lies between 4 and 7, or non-acidic if the pH > 7.

Adding impedance monitoring to standard pharyngeal or pharyngoesophageal pH monitoring improves its sensitivity and false-negative rates [30,51]. Patients with typical LPR signs and symptoms, or appropriate RSI or RFS scores, and one or more proximal reflux episodes in MII-pH are considered by most investigators to be patients with LPRD [36,56,76]. Furthermore, MII-pH monitoring may also help diagnose GER in addition to LPR, as these presumably different entities frequently coexist.

MII-pH has, nevertheless, several limitations. Firstly, it is an invasive and time-consuming examination, and placement of the probe may sometimes require general anesthesia in pediatric patients. Especially in the pediatric population, the proper location of the upper channel is often undone because of the varying, age-dependent length of the esophagus. Moreover, young children might need to be hospitalized for 24 h to prevent them from pulling the tube out. Additionally, analysis and interpretation of the recordings are also time-consuming and require considerable expertise. Last but not least, data on MII-pH monitoring in pediatric patients with LPR and normal children, per age, are lacking. Due to the relatively invasive nature of the exam, data from healthy infants or children is not easy or convenient to be obtained ethically. Diagnostic criteria are adopted from adults and have not been validated in the various pediatric age subgroups; there is, therefore, a lack of standardized diagnostic criteria for the specific population [54,56].

#### 3.6.3. 24-h Pharyngeal pH Monitoring

This technique has several advantages compared with the classic pH-monitoring or MII-pH, but it also has significant disadvantages. Compared to MII-pH, 24-h pharyngeal pH monitoring is relatively non-invasive. It is well tolerated by children as young as seven months old, giving it a very good feasibility and practicality prospect [32]. It can be easily placed in the oropharynx in the outpatient clinic, does not need esophageal probe insertion, and does not need X-ray imaging verification of probe placement. It does not usually cause dysphagia, gagging, or discomfort in the throat with swallowing [37,52]. Because of wireless signal transmission, the recorder may be up to 4 m from the patient and can be placed safely on a bedside table at night [57]. An alternative technique would be double probe pH monitoring, with the distal probe in the esophagus and the upper probe just above the upper esophageal sphincter. The advantage would be that it can concurrently assess for GER and LPR, but on the other hand, it bares all the disadvantages of probe insertion inside the esophagus.

The main disadvantage of 24-h pharyngeal pH monitoring compared to MII-pH monitoring is that it can detect only acidic reflux and not weakly acid or non-acidic reflux, which has also been implied in the pathogenesis of LPR [30,36]. Therefore, pH monitoring without MII might miss the correct diagnosis, especially in infants and young children. Secondly, as with MII-pH, there is currently no consensus for interpreting the results. Due to the lack of pediatric normative values, many authors have adopted criteria from adult pH-metry. Some investigators accept a pH drop < 5.0 or 4.0 in the pharyngeal sensor as an indicator of clinically significant LPR. However, the number of episodes regarded as pathological is still debated. Some believe that more than three episodes of LPR must be present within 24 h for it to be pathological [59], whereas others consider a single episode pathological [32]. Other investigators use the Ryan score, which considers the number of reflux episodes, the duration of the longest episode, and the percentage of time below the respective threshold. Most monitoring systems generate a Ryan score automatically. A Ryan score of more than 9.4 in the upright position or 6.8 in the supine position is considered pathological [32,37].

#### 3.6.4. Pepsin A as a Biomarker

Over the past few years, several studies emphasized pepsin’s role as a promising non-invasive marker of LPR. Pepsin A, which is present in all refluxates regardless of pH, can be measured in saliva, nasal and laryngeal lavage samples, tears, middle ear fluid, and pharyngolaryngeal mucosa [11,13,21,23,43,44,46]. The non-invasive nature of salivary pepsin sampling, in particular, defines its high clinical relevance and potential in the pediatric setting.

Pepsin demonstrates an association with MII-pH monitoring results; however, it is not always present in MII-pH-diagnosed LPR patients and may be present in patients with symptoms of LPR but with negative impedance studies [33,95]. In a study concerning infants with laryngomalacia, salivary pepsin was detected in 81% of patients versus 12.5% of controls [96]. In another study, salivary pepsin was detected in 83% of children with corrected esophageal atresia, and this was significantly associated with GERD symptoms or wheezing [26]. Moreover, pepsin testing on BAL fluid samples seems to have a high positive predictive value and may be a feasible means to identify pediatric patients with chronic respiratory symptoms due to aspiration associated with LPR [53]. In another study, investigators showed that preterm infants that had higher concentrations of pepsin in tracheal aspirates were at greater risk for more severe bronchopulmonary dysplasia than patients with lower concentrations [97].

A recent study in the adult population found that salivary pepsin could be an alternative tool to diagnose LPR in an office-based setting, with a specificity of 86.2% and sensitivity of 41.5% when using a cut-off value of 216 ng/mL [95]. Other authors reported a specificity of 98.2% for reflux disease when salivary pepsin had a concentration of more than 210 ng/mL [98]. The reliability of the pepsin measurement appears to depend on various factors, including the utilized technique (ELISA vs. lateral flow device/Peptest™ vs. Western blot), the cut-off point used, the number of positive samples for deeming a result positive, and the timing and site of sample collection [41,45,98,99,100,101]. Pepsin’s concentration in saliva is probably different in the pediatric population and may also vary considerably in the various pediatric age subgroups. Future investigations need to address all these parameters to clarify the best methodology to use salivary pepsin in the diagnostic process of pediatric LPR.

### 3.7. Management

Generally, the typical approach to managing pediatric LPRD includes behavioral modifications in all patients and pharmacological treatment in those that do not respond to the former. Surgery is reserved only in exceptional cases that prove refractory to maximal pharmacotherapy. Many infants and children do, fortunately, respond to conservative measures such as diet and lifestyle modifications, and these measures are the most logical, cost-effective empirical option for patients with mild to moderate LPRD [102].

#### 3.7.1. Behavioral Modifications

In infants and children, behavioral modifications for LPRD typically center on the following actions:

1. Altering food composition. Thickening of milk and feedings with starch, cereals, carob bean thickeners, or xantham gum improves laryngeal sensation and overall swallowing function and may help in reducing regurgitations and the signs and symptoms of reflux disease as suggested by two systematic reviews of the relevant literature [103,104].
2. Breastfeeding. Infants that breastfeed have less GER and regurgitation [105,106].
3. Use smaller but more frequent feedings while maintaining an appropriate total daily amount of formula or breastmilk [107].
4. Avoidance of feeding just before bedtime.
5. Adjusting the child’s or mother’s (in case of breastfeeding) diet to eliminate substances that increase the number of transient relaxations of esophageal sphincters, such as chocolate, caffeine, alcohol, mint, peppermint, fried and fatty foods, etc.
6. Avoidance of acidic or spicy meals or beverages, such as citrus fruits and juices or tomatoes and tomato-based foods. Carbonated beverages may cause gastric bloating and should also be avoided. Keeping a diary of food/beverage intake is often helpful.
7. Treating functional constipation. Treatment of constipation in children with the use of a high-fiber diet, behavioral education, or laxatives can reduce acid reflux and improve reflux symptoms [34,58].
8. Elimination of secondhand smoke. Tobacco is a common cause of GER/LPR in adults, and environmental smoking was also implied to cause reflux in infants and neonates [108].
9. Keeping an infant in a relatively vertical position for at least 30 min after feeding.
10. Elevating the head of the bed may be used in preschool and school-aged children but is not recommended in infants and children younger than two years of age, as it is uncertain that it helps reduce reflux, but, most importantly, because of the risk of suffocating, as it may cause the baby to slide down to the foot of the cot or under the beddings [3,104,109,110].
11. Sleep positioning may provide further benefits to infants; however, the ideal position remains a subject of debate [66,111]. Importantly, because of sudden infant death syndrome (SIDS) concerns, the supine sleeping position is considered safer, as the prone and side sleeping positions increase the risk of SIDS significantly [109,112]. Currently, the American Academy of Pediatrics advises that the supine position is recommended in infants with GER. Alternative positioning during sleep is considered reasonable only exceptionally in infants with specific upper airway disorders in which the risk of death from GERD/LPRD may outweigh the risk of SIDS [109].

Of note, a subset of infants with cow’s milk protein allergy experience regurgitation and vomiting indistinguishable from that associated with LPR or GER [3]. Therefore, a 2 to 4-week trial of extensively hydrolyzed protein formula or restricting all dairy products from the mother’s diet in a breastfed infant is worthwhile in infants who have not responded to maximal, conventional, or non-pharmacologic LPR therapies. The trial is considered positive if apparent regurgitation or vomiting decreases significantly in frequency within 4 weeks, and reintroduction causes recurrence of symptoms.

#### 3.7.2. Pharmacotherapy

When behavioral modifications prove insufficient in resolving symptoms of LPR or improving related conditions, medical therapy is the next step to explore. As with adults, the mainstay of pharmacological treatment is considered to be proton pump inhibitors (PPIs). Histamine-2 receptor antagonists, once a mainstay in treatment, have now become second-line agents, as they provide a much shorter duration of action (4–8 h) compared to PPIs, less inhibition of meal-stimulated acid secretion, and worse control of symptoms [55]. In children, they are sometimes used to either help wean patients off PPIs or to supplement PPI therapy by administrating a dose at night to provide nocturnal acid suppression [102,113]. Promotility agents, such as metoclopramide, erythromycin, and domperidone, have been studied in the past, but due to their lack of predictable efficacy and safety, they are nowadays seldom used [66,88].

##### Proton Pump Inhibitors

PPIs bind covalently to acid pumps of the gastric mucosa and provide the greatest efficacy when taken 30 min before a meal [114]. Administration after a feeding, or more than an hour before, significantly limits acid suppression efficacy [115]. PPIs are currently the most prescribed drugs for reflux in the pediatric setting [19]. FDA-approved agents for pediatric use include omeprazole, lansoprazole, esomeprazole, and pantoprazole. For infants, only esomeprazole and omeprazole are FDA-approved, and, in fact, these are indicated for erosive esophagitis due to acid-mediated GERD only [116].

Due to the lack of well-designed randomized placebo-controlled trials and the heterogeneity in outcomes and methods, the efficacy of PPIs for GERD, and especially LPRD, remains questionable in the pediatric population. Two systematic reviews demonstrated conflicting evidence for the efficacy of PPIs in pediatric reflux disease, especially in infants [117,118]. However, evidence suggesting rough equivalency between PPIs and placebo should not be interpreted as supporting that patients with true acidic LPR could not benefit from PPIs as it probably reflects the inability of studies to include only patients with acidic reflux and the excess heterogeneity among studies regarding objective or subjective diagnostic methods, inclusion and exclusion criteria, variability of treatment protocols, and differences in outcome measures [2].

Given this paucity of the existing literature on the pediatric population, there is restricted evidence to guide optimal dosing and duration for pediatric LPRD treatment [19]. However, pharmacokinetics and data from adults support the superiority of administrating PPIs twice vs. once daily, and four months vs. two months in duration, as it provides more time for mucosal healing and regeneration [119]. In infants, however, given the uncertainty in efficiency, physicians should avoid the chronic prescription of PPIs with regard to their long-term side effects concerning calcium and magnesium metabolism, bone mineral density, B12 deficiency, infections including Clostridium difficile colitis, and upper and lower respiratory tract infections [3,19,120,121]. Because in most infants, LPR spontaneously resolves or significantly improves within months, behavioral modifications should be kept after PPIs discontinuation, and another course of PPIs could be prescribed, should such modifications prove inadequate in preventing symptoms from recurring.

##### Alginates

As highlighted above, the universal administration of PPIs in the LPRD therapeutic strategy is problematic since they are less effective on nonacid or weakly acidic LPRD variants, which appear to be quite common in the pediatric population. Moreover, given that trypsin is an alkaline protease and requires alkaline pH to become and remain active, administering high doses of PPIs may even be associated with the deterioration of complaints in these patients [2]. Identification of acidic versus non-acidic LPRD by means of MII-pH is crucial to selecting the optimal pharmacological treatment, and this fact may explain the mixed results concerning PPIs effectiveness and the high rates of patients considered PPI-resistant in previous studies that did not use MII-pH for diagnosis.

Alginic acid derivatives (alginates) are a logical option in nonacid or mixed reflux, alone or in combination with PPIs. Alginates form a raft floating over the gastric contents, preventing gaseous or liquid reflux into the esophagus and laryngopharynx. Alginates can be maintained inside the stomach for up to 4 h and may also form a protective film on the mucosa of the esophagus and upper aerodigestive tract that preserves functional barrier integrity [122]. They are usually prescribed three to four times daily (after main meals and at bedtime) when used alone or one to four times daily when used in conjunction with PPIs.

Several studies have demonstrated alginate’s superiority over controls in treating adult LPRD patients [123,124]. According to a recent article about the adult population, liquid alginate suspension prescribed four times daily proved effective in treating symptoms of LPRD. At the same time, co-prescription with high-dose PPIs offered no additional benefit [124]. Concerning the management of reflux in infants, alginates were shown to be more effective compared to no treatment and more or at least equally effective compared with behavioral modifications (milk thickeners) [125,126]. They were also shown to significantly reduce the number and extension of acidic and non-acidic reflux episodes in MII-pH monitoring studies in infants [127].

#### 3.7.3. Anti-Reflux Surgery

Patients that do not respond to behavioral modifications and maximal pharmacotherapy should first be assessed for compliance with treatment recommendations. Second, a thorough revaluation by all involved subspecialties (pediatric gastroenterologists, otorhinolaryngologists, allergologists, and surgeons) is also advisable in order to explore other possible diagnoses or contributors to persistent LPR such as achalasia, eosinophilic esophagitis, cow’s milk protein allergy, Zenker diverticulum, tracheoesophageal fistulas, laryngeal cleft, diffuse esophageal spasm, absent esophageal peristalsis, hypercontracting esophagus, hiatal hernia, and gastroparesis. Those who are truly treatment-resistant and continue to have potentially life-threatening complications, such as severe apneic episodes, bradycardia, and recurrent pneumonia, or failure to thrive despite aggressive medical treatment (especially those having an underlying neurological impairment or gastroenterological evaluations demonstrating severe hiatal hernias), may benefit from surgical intervention. The most common surgical procedures used are the laparoscopic Nissen, Thal, or Toupet fundoplications that aim at reconstructing a competent lower esophageal sphincter by repairing the hiatal defect and performing an anti-reflux wrap of the fundus (total or partial) around the lower esophagus [128,129,130,131].

### 3.8. Summary and Future Perspectives

Recently, the available evidence in pediatric LPR has been gradually increasing. However, its strength remains relatively low, as the vast majority of the studies listed in Table 1 comprise case-control or cohort studies with substantial heterogeneity in their population, inclusion/diagnostic criteria, and outcome measures.

There is still no recent epidemiologic study regarding the incidence and prevalence of pediatric LPRD using objective diagnostic tools. Moreover, no study estimates the rate of various symptoms and findings on a large cohort of (objectively) confirmed LPR patients in the various pediatric age groups. Further investigations are needed to establish the mechanisms underlying the pathogenesis of laryngopharyngeal damage in children. This could also help develop novel therapeutic options for pediatric LPR.

The most commonly utilized assessment tools include structured questionnaires of relevant symptoms, endoscopic evaluation of the larynx, 24 h pharyngeal pH monitoring, and 24 h MII–pH monitoring. Clinical tools used in the assessment of the disease in children are adopted from adult-orientated tools. They are, therefore, suboptimal as they do not take into consideration many age-specific symptoms and extra-laryngeal findings. Even more importantly, these tools have not been validated for use in pediatric populations. Development and validation of novel pediatric-orientated assessment tools are highly needed.

Regarding diagnostic methods, fortunately, an increasing number of authors have been using MII-pH monitoring (Table 1); however, data concerning normative values in the various pediatric age subgroups are lacking, and there is still no consensus regarding the diagnostic criteria. Further studies are urgently needed for the standardization and proper validation of MII-pH monitoring in children, ultimately leading to the emergence of consensual diagnostic criteria. Better delineating the role of pepsin as a biomarker in pediatric patients may allow more options in non-invasive, in-office testing and additional objective diagnostic parameters.

Overall, evidence able to guide the management of LPRD in the pediatric population is still weak, and there is high heterogeneity among controlled trials leading to inconclusive findings about the superiority of PPIs over placebo. Despite recent advances and increased awareness of the impact of the weakly acidic and non-acidic reflux variants of pediatric LPR, pharmacological treatment is still primarily centered on prescribing PPIs. Further studies are needed to delineate the role of alginates in the specific age group, and future research should explore novel agents directly targeting pepsin or its receptors in the laryngopharyngeal mucosa.

## 4. Conclusions

In conclusion, with regard to the high level of controversy about the epidemiology, assessment, diagnosis, and management of pediatric LPRD, further high-quality original research covering all these aspects is urgently needed. In daily clinical practice, based on the data accumulated so far, involved specialists should, for the moment, focus on defining and utilizing a multiparameter, test battery diagnostic approach, starting from age-appropriate questionnaires and parent- or patient-reported outcome measures, flexible nasopharyngolaryngoscopic findings, and, perhaps, salivary pepsin, and escalating to 24-h pharyngeal pH and MII-pH monitoring whenever appropriate and available. For the time, a personalized, step-wise therapeutic plan is the most reasonable management approach. Mild to moderate and uncomplicated LPRD should initially be treated with behavioral changes. In severe or nonresponsive cases, after at least four weeks of such implementations, the attending healthcare providers should use a personalized approach with a combination of behavioral changes and PPIs or alginates (or both) according to the patient’s reflux profile in MII-pH monitoring whenever available. Surgical treatment options could be considered in the most severe cases when potentially life-threatening symptoms persist despite maximal medical therapy. Further major steps in the research of pediatric LPR require well-conducted, large, multicenter, randomized controlled studies with standardized protocols and uniformity in diagnostic procedures and criteria.

## Figures and Tables

**Table 1:** A summary of all original articles on pediatric LPR that were published in the past 10 years (2012–2021).

Study	Design	Population (Age Range)	Diagnostic Modality	Scope/Subject	Conclusions/Clinical Relevance
Mahoney et al. (2021) [20]	Prospective	43 children with chronic respiratory symptoms (1–18 y.o.)	MII-pH	Evaluate the metabolite profile in BAL fluid from children with suspected LPR and assess the impact of reflux treatment on these metabolites	The study explored the impact of reflux and PPIs on the lungs and provided a foundation for future studies for novel potential biomarkers of LPR
Samuels et al. (2021) [21]	Prospective	30 pediatric subjects undergoing tympanostomy tube placement for COME (0–12 y.o.)	Patients were not diagnosed with LPR, but pepsin in middle ear was thought to arise from LPR	Investigate the association of pepsin with middle ear inflammatory signaling	Pepsin was observed in middle ear fluid in 77% of patients, and this correlated with increased IL-6 and -8, neutrophil elastase, and mucin 5B; therefore, pepsin may contribute to inflammatory signaling
Ugras et al. (2021) [22]	Retrospective	52 children that underwent gastroesophagoscopy and had LPR symptoms (5–17 y.o.)	RSI and RFS	Explore associations between RFS, RSI, and the severity of esophagitis on histopathology	A correlation between RFS and histopathological findings was found
Tumgor et al. (2021) [16]	Prospective	44 children requiring adenotonsillectomy and 22 children without reflux symptoms as a control group (>3 y.o.)	GERD diagnosed by esophageal pH monitoring and histopathological evaluation for esophagitis.	Determination of GERD incidence in children requiring adenotonsillectomy due to adenotonsillar hypertrophy	Reflux was detected in 72.7% of children requiring adenotonsillectomy vs. none in the control group
Lei et al. (2021) [23]	Prospective	65 children diagnosed with COME (2–14 y.o.)	No LPR-diagnosed subjects were included, but pepsin in the middle ear was thought to derive from LPR	Correlation between pepsin A and DNA from bacteria with IL-8 and TNF-a in the effusion of children with COME	The study showed that bacterial infection and LPR might act in synergy in the pathogenesis of COME
Singh et al. (2020) [24]	Prospective	50 children with recurrent tonsillitis (6–18 y.o.)	RSI and RFS	Detection of H. pylori and LPR in patients with recurrent tonsillitis	LPR was not found to be a significant factor in the colonization of tonsils by H. pylori
Dziekiewicz et al. (2020) [15]	Prospective	38 subjects with adenoid hypertrophy (3–15 y.o.)	MII-pH	Estimate the frequency of GERD among children with adenoid hypertrophy	First study to use MII-pH to assess GERD in children with adenoid hypertrophy. Only 13.2% of patients had GERD
Mantegazza et al. (2020) [25]	Prospective	197 children with suspected LPR (0–17 y.o.)	MII-pH	Investigation of the reliability of RFS and RSI in assessing pediatric GERD/LPR	RSI and RFS were not found accurate in predicting pediatric GERD/LPR
Upendran et al. (2020) [26]	Prospective	18 children with repaired esophageal atresia (6–16 y.o.)	Pepsin A with Peptest™ and ELISA	Investigate pepsin in exhaled breath condensate and saliva as a potential non-invasive marker of reflux aspiration in children with esophageal atresia	Salivary pepsin was detected in a large proportion of subjects, and this was associated with GERD symptoms or wheezing, substantiating a role for pepsin as a probable non-invasive marker of reflux aspiration
Wertz et al. (2020) [27]	Retrospective	163 children with LPR (2.5–17 y.o.)	Clinical diagnosis based on symptoms and endoscopic findings	Laryngologic/dysphonia assessment (pVHI, acoustic analysis, laryngeal endoscopy) in LPR-diagnosed children	Pediatric patients with clinically diagnosed LPR have pVHI, jitter, and shimmer scores that are comparable to those of pediatric patients with dysphonia due to other etiologies
Košec et al. (2020) [28]	Retrospective	89 children who presented with extra-esophageal GERD symptoms (NA)	24-h double-probe pH monitoring for both GER and LPR diagnosis	Evaluate the prevalence of extraesophageal symptoms and the reliability of a novel screening score	52 patients were diagnosed with GERD, and out of them 50 had LPR. Obesity was frequent in patients placed in higher risk groups for a positive GERD diagnosis
Maholarnkij et al. (2020) [29]	Prospective	15 children with esophageal atresia (1.4–12.9 y.o.)	MII-pH	To explore associations of symptoms (esophageal and extraesophageal) and MII-pH findings in children with esophageal atresia	Prevalence of GERD in children with atresia was high and MII-pH showed a high diagnostic value in the specific population
Lee et al. (2020) [18]	Retrospective	5747 children diagnosed with GERD within the first year of life and 14,877 children without GERD serving as controls (0–1 y.o.)	NA	Evaluate otologic outcomes in children with a diagnosis of GERD using a large pediatric hearing database	GERD diagnosed within the first year of life is associated with otologic issues including hearing loss, otitis media, eustachian tube dysfunction, and need for tympanostomy tubes
Plocek et al. (2019) [30]	Prospective	23 children with suspected LPR (3–16 y.o.)	24-h pharyngeal pH monitoring and MII-pH	Assess the correlation between acid reflux episodes recorded by pharyngeal pH monitoring and GER detected via MII-pH	Findings show that the efficacy of the exclusive application of pharyngeal pH monitoring for LPR diagnosis is uncertain
Formánek et al. (2019) [14]	Prospective	11 children with JORRP (4–14 y.o.)	Presence of pepsin in biopsy specimens	Explore associations between LPR and JORRP	Pepsin was detected in 45.5% of specimens, implying LPR as a probable risk factor for JORRP
Galli et al. (2019) [31]	Prospective	35 children (0–16 y.o.)	RSI and RSF	Evaluate the value of NBI in identifying LPR signs in the pediatric population	NBI could help improve the identification of endoscopic signs of LPR
Wlodarczyk et al. (2019) [32]	Prospective	68 children (3–18 y.o.)	24-h pharyngeal pH monitoring	Correlate laryngoscopic findings with RFS and 24-h pharyngeal pH monitoring in children with voice disorders	Vocal fold edema, laryngeal edema, and posterior commissure mucosal hypertrophy were found to be important determinants of LPR
Klimara et al. (2019) [33]	Prospective	16 laryngomalacia children and 16 controls (0–2 y.o.)	Salivary pepsin A	Explore associations between salivary pepsin and the presence and severity of laryngomalacia	Pepsin was detected in 13 patients with laryngomalacia vs. 2 controls, implying a probable role for LPR in the pathogenesis of laryngomalacia
Baran et al. (2017) [34]	Prospective	55 children with functional constipation and suspected GERD (5–18 y.o.)	GERD diagnosed with esophageal 24-h pH monitoring	Explore the impact of constipation treatment on GERD symptoms and 24-h pH monitoring	Treatment of constipation can improve the reflux symptoms and abnormal acid reflux
Siupsinskiene et al. (2017) [35]	Prospective	97 patients undergoing tonsillectomy, of which 36 were children (<18 y.o.)	RSI and RFS	Identify H. pylori infection in tonsillar tissue from patients undergoing tonsillectomy and explore its relationship with LPR symptoms and laryngoscopic signs	56.5% of patients with chronic tonsillitis had H. pylori, and this was associated with signs of vocal fold edema, diffuse laryngeal edema, and hypertrophy of the posterior commissure
Luebke et al. (2017) [13]	Prospective	15 children undergoing airway surgery; 10 with laryngomalacia and 5 controls (0–3 y.o.)	Pepsin A in supraglottic lavages and arytenoid biopsies	Explore correlations between the presence of pepsin in the airways of laryngomalacia patients vs. controls	Pepsin in supraglottic specimens demonstrated an association with laryngomalacia, supporting a probable pathogenetic role for LPR
Górecka-Tuteja et al. (2016) [6]	Prospective	28 children with COME (7–10 y.o.)	MII-pH	Detect and characterize reflux events in COME patients	LPR was noted in 68% of children with COME implying a role for LPR in the pathogenesis of the disease
Pavic et al. (2016) [36]	Prospective	104 children with suspected LPR (0–18 y.o.)	MII-pH	Determine MII-pH characteristics and assess correlation with RFS and RSI	Both acid and non-acid reflux appeared to have a significant role in the pathogenesis of pediatric LPR
Mesallam et al. (2016) [37]	Prospective	26 children with airway-related problems (0–16 y.o.)	24-h pharyngeal pH monitoring	Study the feasibility of 24-h pharyngeal pH monitoring in the outpatient setup and explore correlations with airway-related problems	85% of the patients tolerated the pH probe insertion and completed the exam. Laryngomalacia and subglottic stenosis were frequently reported in LPR patients (77%)
Singendonk et al. (2016) [38]	Retrospective	30 infants (0–1 y.o)	Subjects were not LPR patients	Determine the reliability of the RFS-I (RFS for infants) for flexible versus rigid laryngoscopy in infants	The reliability of the RFS-I was moderate with flexible and rigid laryngoscopy. The RFS-I is not suitable for detecting signs or guiding treatment of LPR in infants
Kim et al. (2016) [39]	Prospective	84 patients with tonsillar hypertrophy of which 54 children (4–16 y.o.)	Presence of pepsin in tonsillar tissue was considered to derive from LPR	Explore the role of pepsin in the pathogenesis of tonsillar hypertrophy	Pepsin may play a role as it was detected in the hypertrophic tonsils and its presence correlated with damaged epithelium and expression of various pro-inflammatory mediators
Duncan et al. (2016) [40]	Prospective	116 children undergoing MII-pH (NA)	MII-pH	Determine if rates of hospitalization are affected by reflux burden, even after adjusting for aspiration risk	Even in aspirating children, reflux burden was not found to increase the risk of hospitalization
Dy et al. (2016) [41]	Prospective	50 children (1–19 y.o.)	MII-pH and salivary pepsin using the PepTest™	Compare salivary pepsin with pH-MII and endoscopy and determine its sensitivity	Salivary pepsin was found to show a low sensitivity for predicting GERD in children
Doğru et al. (2015) [42]	Prospective	31 children with OME and 19 with no effusion (2–15 y.o.)	Pepsinogen levels and H. pylori in middle ear aspirates were considered indicative of LPR	Explore associations between otitis media with effusion and LPR in children	In children with OME, pepsinogen levels in the middle ear were significantly higher than in the serum. 19% of patients were positive for H. pylori in middle ear aspirates
Önal et al. (2015) [12]	Prospective	51 children with recurrent episodes of URTI, recurrent OME, or sinusitis (3–15 y.o.)	Symptoms questioning and endoscopic findings for LPR diagnosis and histology for esophagitis/GERD diagnosis	Explore associations between LPR and GERD with specific symptoms or clinical findings	The likelihood of the occurrence of esophagitis was found to be increased in the presence of recurrent OME and postglottic edema, irrespective of the presence of reflux symptoms
Salturk et al. (2015) [17]	Prospective	60 children undergoing tonsillectomy, of which 18 with LPR and 42 controls (age range: NA)	RSI and RFS	Calculate rates of complications post-tonsillectomy in LPR patients vs. controls	LPR is a probable risk factor for complications following tonsillectomy
Iannella et al. (2015) [11]	Prospective	20 LPR-diagnosed children and 20 controls (1–15 y.o.)	MII-pH	Identify the presence or absence of pepsin in tears from children with LPR	20% of LPR subjects showed pepsin in tears, implying a role in the pathogenesis of sinusitis or dacryostenosis.
Formánek et al. (2015) [43]	Prospective	24 children with COME (1–7 y.o.)	24-h pharyngeal pH monitoring and pepsin in middle ear fluid	Compare 3 modalities of detecting LPR in COME patients (pharyngeal pHmetry, Peptest^®^ to detect pepsin in ear fluid, and pepsin in adenoids by immunochemistry)	pH monitoring was pathological in 13/21 (61.9%) children. Pepsin in the middle ear fluid was present in 5/21 (23.8%) children; these 5 patients had more severe LPR in pH monitoring
O’Reilly et al. (2015) [44]	Prospective	129 children with COME, altogether 199 ear samples (0–16 y.o.)	No LPR-diagnosed subjects were included, but pepsin in the middle ear was thought to derive from LPR	Investigate the correlation of pepsin (measured by immunoassay) with cytokines, bacterial infection, and clinical outcomes	Levels of pepsin were correlated with IL-8 levels and the need for further tube placements; thus, LPR may play a role in the pathogenesis of COME
Formánek et al. (2015) [45]	Prospective	44 children with COME, altogether 59 ears (1–7 y.o.)	No LPR-diagnosed subjects were included, but pepsin in the middle ear was thought to derive from LPR	Investigate whether Peptest™ could be used to identify pepsin in the middle ear fluid children with COME	Pepsin was detected in 19/59 (32.2%) of middle ear specimens, implying a role for LPR in the pathogenesis of COME
Nation et al. (2014) [10]	Retrospective	63 children with rhinorrhea, nasal congestion, and chronic cough (0–10 y.o.)	Histology showing esophagitis was considered diagnostic for GERD	Explore associations between pediatric CRS and GERD in children with symptoms of rhinorrhea, nasal congestion, and chronic cough	Over 40% of all patients had positive gastroesophageal biopsies
Luo et al. (2014) [46]	Prospective	48 children with COME, 50 with adenoid hypertrophy, and 30 controls (2–8 y.o.)	Pepsin and pepsinogen concentrations in ear fluid and plasma	Explore the relationship between LPR and COME	Pepsin and pepsinogen were increased in middle ear effusion; thus, LPR may be involved in the pathogenesis of COME
Baudoin et al. (2014) [47]	Retrospective	89 children (1–18 y.o.) with suspected LPR	24 h double probe pH monitoring	Development of new probability score based on symptoms, local findings, and comorbidities	Presented an attempt to classify diagnosis likelihood by defining groups at higher risk
Van der pol et al. (2014) [48]	Retrospective	52 infants (0–1 y.o.)	Clinical diagnosis based on flexible laryngoscopy	Development and validation of a modified edition of RFS for infants	The new tool reached only moderate interobserver agreement with a highly variable intraobserver agreement
Katra et al. (2014) [49]	Prospective	30 children with adenoid hyperplasia (2–8 y.o.)	MII-pH	Investigate associations between LPR and H. pylori in adenoid hyperplasia	Patients with H. pylori had significantly more reflux episodes; thus, LPR may play a role in the transmission of H. pylori to adenoids and contribute to hyperplasia
Aydin et al. (2014) [50]	Prospective	32 patients with adenoid hypertrophy (4–13 y.o.)	24-h dual probe pH monitoring	Investigate the role of LPR and H. pylori colonization in adenoid hypertrophy	Only 5 patients had LPR, none of which had a H. pylori positive adenoidectomy sample
Rosen et al. (2014) [51]	Prospective	112 children with chronic cough and wheezing (1–16 y.o.)	MII-pH and gastrointestinal endoscopy	Evaluate the role of reflux testing (endoscopy and pH-MII) in the specific group of patients	There is a high yield to reflux testing in children with chronic cough and wheezing, as 58% had either an abnormal MII-pH, or endoscopy
Andrews et al. (2013) [52]	Retrospective	63 children with suspected LPR (0–17 y.o.)	24-h pharyngeal pH monitoring	Comparison of histologic findings from the post-cricoid region versus pharyngeal pH probe results	24 h pharyngeal pH monitoring was well tolerated and with no complications, representing a useful tool in confirming clinical suspicion
Abdel-aziz et al. (2013) [9]	Prospective	50 children with COME (1–10 y.o.)	24 h dual-probe pH monitoring and pepsin A in middle ear effusions	Evaluate the clinical role of pepsin and LPR in children with COME	There was a significant correlation between the level of pepsin in the effusions and the number of reflux episodes implying a role for LPR in the pathogenesis of COME
Kelly et al. (2013) [53]	Prospective	76 patients with chronic pulmonary disease, of which 65 study patients and 11 controls (0–24 y.o./only two were >18)	Pepsin A detection by Western blot in BAL	Determine the prevalence of aspiration-associated extra-esophageal reflux in patients with chronic respiratory symptoms by detecting the presence of pepsin in BAL specimens	72% of study group had (+) BAL vs. 0% of controls. Detection of pepsin in BAL can serve as a biomarker for aspiration-associated extra-esophageal reflux disease
Kilic et al. (2013) [54]	Prospective	50 children with persistent asthma (7–17 y.o.)	24-h double probe pH monitoring	Explore correlations of LPR and GERD diagnosis with RSI and RFS, and status of asthma (controlled vs. uncontrolled)	RSI and RFS did not seem reliable in diagnosing LPR and GER in children. There was no association between asthma control status and LPR or GERD
Ummarino et al. (2012) [55]	Prospective	35 children with extraesophageal symptoms and positive MII-pH (0–15 y.o.)	MII-pH	Compare treatment outcomes of 3 months ± another 3 months if not symptom-free with omeprazole versus ranitidine	The efficacy of omeprazole was superior to that of ranitidine in these children
Greifer et al. (2012) [56]	Retrospective	63 children with extraesophageal symptoms (0–17 y.o.)	MII-pH	Explore associations of extraesophageal symptoms with pathological acid or nonacid reflux	No association was demonstrated between the extraesophageal signs and symptoms and pathological reflux in MII-pH
Banaszkiewicz et al. (2012) [57]	Prospective	21 children with difficult-to-treat asthma (7–17 y.o.)	24-h pharyngeal pH monitoring	Assess the prevalence of LPR in children with difficult-to-treat asthma	LPR was diagnosed in 61.9% of children. There was a correlation between LPR and the degree of asthma control
Baran et al. (2012) [58]	Prospective	38 children with functional constipation and 40 children with suspected GERD (4–16 y.o.)	24-h esophageal pH monitoring	Investigate the frequency of GERD in children with functional constipation	GERD should be considered in the treatment and monitoring of patients with functional constipation.
Ozmen et al. (2012) [59]	Retrospective	49 children with suspected LPR (1–16 y.o.)	24-h double probe pH monitoring and/or scintigraphy	Assess the laryngoscopic findings in children diagnosed with LPR or GERD	12 of 30 patients diagnosed with LPR or GERD had a positive laryngeal finding. Laryngoscopy showed a 40% sensitivity and 50% specificity in the diagnosis of LPR/GERD

MII-pH: Multichannel intraluminal impedance-pH monitoring. BAL: Bronchoalveolar lavage. LPR: Laryngopharyngeal reflux. PPIs: Proton pump inhibitors. COME: Chronic otitis media with effusion. RSI: Reflux symptom index. RFS: Reflux finding score. GERD: Gastroesophageal reflux disease. ELISA: Enzyme-linked immunosorbent assay. pVHI: Pediatric Voice Handicap Index. JORRP: Juvenile onset recurrent respiratory papillomatosis. NBI: Narrow-band imaging. OME: Otitis media with effusion. URTI: Upper respiratory tract infection. NA: Information not available.

## Data Availability

Data sharing is not applicable to this article.
